# 9-ING-41, a small molecule inhibitor of GSK-3beta, potentiates the effects of anticancer therapeutics in bladder cancer

**DOI:** 10.1038/s41598-019-56461-4

**Published:** 2019-12-27

**Authors:** Hiroo Kuroki, Tsutomu Anraku, Akira Kazama, Vladimir Bilim, Masayuki Tasaki, Daniel Schmitt, Andrew P. Mazar, Francis J Giles, Andrey Ugolkov, Yoshihiko Tomita

**Affiliations:** 1 Department of Urology, Molecular Oncology, Graduate School of Medical and Dental Sciences, Niigata University, Niigata, Japan; 2 Kameda Daiichi Hospital, Niigata, Japan; 3 Actuate Therapeutics, Fort Worth, TX USA; 4 Monopar Therapeutics, Wilmette, IL USA

**Keywords:** Cancer, Oncology, Urology

## Abstract

Glycogen synthase kinase-3 beta (GSK-3β), a serine/threonine kinase, has been identified as a potential therapeutic target in human bladder cancer. In the present study, we investigated the antitumor effect of a small molecule GSK-3β inhibitor, 9-ING-41, currently in clinical studies in patients with advanced cancer, in bladder cancer cell lines. We found that treatment with 9-ING-41 leads to cell cycle arrest, autophagy and apoptosis in bladder cancer cells. The autophagy inhibitor chloroquine potentiated the antitumor effects of 9-ING-41 when tested in combination studies. Our findings also demonstrate that 9-ING-41 enhanced the growth inhibitory effects of gemcitabine or cisplatin when used in combination in bladder cancer cells. Finally, we found that 9-ING-41 sensitized bladder cancer cells to the cytotoxic effects of human immune effector cells. Our results provide a rationale for the inclusion of patients with advanced bladder cancer in clinical studies of 9-ING-41.

## Introduction

Bladder cancer is the 9th most common cancer in the world, with 549,393 new cases diagnosed in 2018^1^. Bladder cancer caused 199,922 deaths in 2018, and most of which were caused by metastatic disease^1^. Historically, bladder cancer patients with metastatic disease received a combination therapy of methotrexate, vinblastine, doxorubicin, and cisplatin (MVAC)^2^. Beginning in the 2000s, a gemcitabine and cisplatin regimen was introduced that showed less toxicity than MVAC with no survival advantage^3^. However, treatment with gemcitabine and cisplatin remains palliative and metastatic bladder cancer remains incurable with median survival time from 12 to 15 months^3^. Thus, the identification of new therapeutic agents is of critical importance to improve clinical outcomes for metastatic bladder cancer patients.

Glycogen synthase kinase-3β (GSK-3β) is a serine/threonine protein kinase which was first described as a component of glycogen synthase (GS) regulation through its phosphorylation^4^. A number of published studies suggested GSK-3β as a potential therapeutic target in non-insulin dependent diabetes mellitus, Alzheimer disease, osteoporosis, inflammatory diseases and cancer^5^. In the context of cancer treatment, GSK-3β is well known as a positive regulator of transcriptional activity of NF-κB^6–8^. NF-κB is involved in tumor chemoresistance, neovasucularization, invasion and metastasis^9^. It has been shown that GSK-3β positively regulates NF-κB–mediated survival of cancer cells and that the inhibition of GSK-3β decreases cancer cell survival via suppression of NF-κB–mediated Bcl-2 and XIAP expression in leukemia^7^, pancreatic^8^, and renal cancer cells^10^. We have previously identified GSK-3β as a promising new therapeutic target in bladder cancer^11^.

In this study, we explored the antitumor effects of 9-ING-41, a maleimide-based ATP-competitive small molecule GSK-3β inhibitor, in bladder cancer cells when combined with an array of potentially active therapeutic agents^12^. 9-ING-41 is more selective for GSK-3β than for other 320 related kinases when tested in a kinase screen^13^. Treatment with 9-ING-41 has shown antitumor effects in neuroblastoma^14^, B-cell lymphoma^15^, glioblastoma^16^, ovarian^12^, pancreatic^17^, renal^10^ and breast cancer^18^. Recently, 9-ING-41 has entered the clinic in trials in patients with different types of advanced cancer (https://clinicaltrials.gov/ct2/show/NCT03678883?term=9-ing&rank=1). Here, we demonstrate that treatment with 9-ING-41 enhanced the antitumor effects of chemotherapeutic drugs, and improved the cytotoxic effect of human immune cells in bladder cancer cell lines.

## Results

### 9-ING-41 treatment induces cell cycle arrest and apoptosis in bladder cancer cells

**Figure 1:**
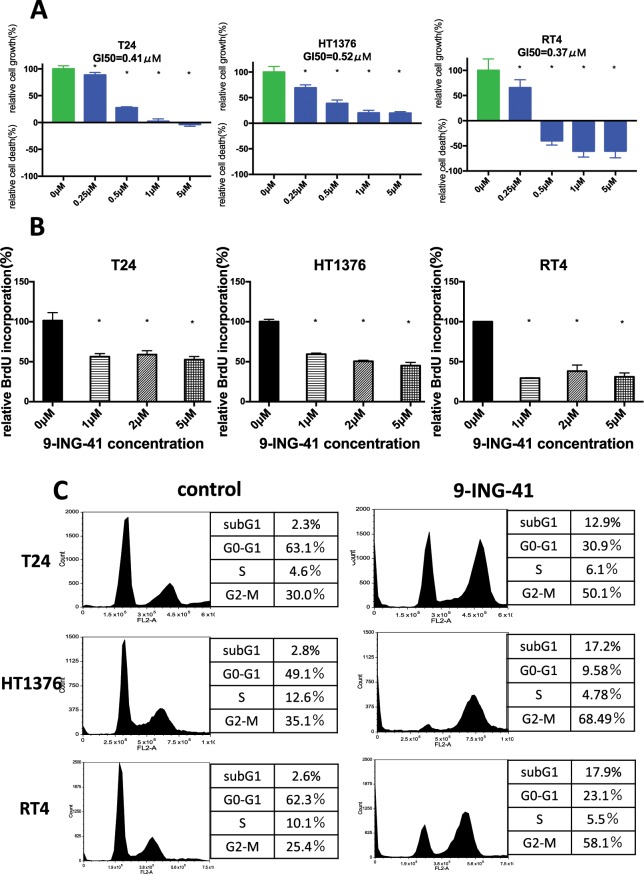
Treatment with 9-ING-41 decreases proliferation and survival of bladder cancer cells. (**A**) Relative cell viability of bladder cancer cells treated with the indicated doses of 9-ING-41 for 96 hours was measured by MTS assay. *P < 0.05 by one-way ANOVA with Tukey post-hoc test. (**B**) BrdU colometric assay was done using bladder cancer cells treated with 9-ING-41 at indicated concentrations for 48 hours. Columns, mean; bars, SD. *P < 0.05 by one-way ANOVA with Tukey post-hoc test. (**C**) Flow cytometry was performed using T24, HT1376 and RT4 bladder cancer cells treated with 10 μM of 9-ING-41 for 72 hours. Cell cycle distribution and fraction of sub-G1, G1, S and G2 population are shown.

**Figure 2:**
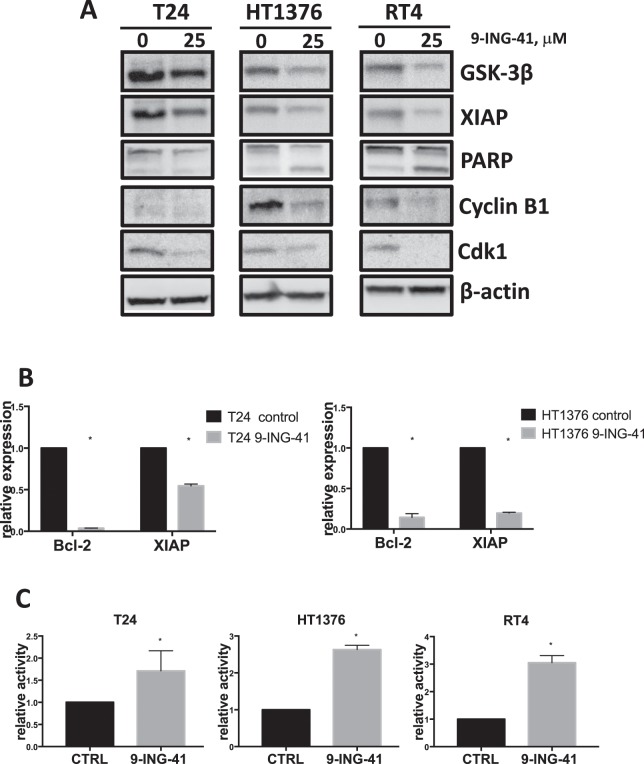
Antitumor effects of 9-ING-41 therapy in bladder cancer cells. (**A**) Bladder cancer cells were treated with indicated concentration of 9-ING-41 for 96 hrs. Post-treatment, whole cell lysate was prepared from the cells, separated by SDS-PAGE (20 μg/well), transferred to PVDF membrane, and immunoblotted as indicated. (**B**) T24 and HT1376 bladder cancer cells were treated with 9-ING-41 for 72 hours and mRNA expression of Bcl-2 and XIAP was analyzed by RT-PCR. Columns, mean; bars, SD. *P < 0.05 by one-way ANOVA with Tukey post-hoc test. (**C**) Activity of caspase-3 was detected in 9-ING-41-treated bladder cancer cells using colorimetric CaspACE Assay System. Columns, mean; bars, SD. *P < 0.05 by one-way ANOVA with Tukey post-hoc test.

We tested 9-ING-41 in T24, HT1376 and RT4 bladder cancer cell lines. 9-ING-41 treatment resulted in a decreased growth of bladder cancer cells at 0.25–1 μM concentrations in a dose-dependent manner with GI_50_ range of 0.4–0.5 μM (Fig. 1A). Moreover, we found a cytotoxic effect of 9-ING-41 in RT4 bladder cancer cells at >0.5 μM concentrations of 9-ING-41 (Fig. 1A). We utilized BrdU incorporation assay to confirm that treatment with 9-ING-41 suppresses proliferation of bladder cancer cells (Fig. 1B). Our finding that 9-ING-41 inhibits proliferation of bladder cancer cells prompted us to examine the effect of 9-ING-41 on cell cycle kinetics. We found cell cycle blockage at G2/M after 24 hours of 9-ING-41 treatment (Fig. 1C), suggesting that GSK-3 inactivation by 9-ING-41 halts progression of mitosis in bladder cancer cells. To investigate the mechanistic effect of GSK-3 inhibitor 9-ING-41 in the blockage of cell cycle in bladder cancer cells, we examined the expression of G2/M regulatory proteins Cdk1 and Cyclin B1 in 9-ING-41-treated cells. We found that expression of Cdk1 and Cyclin B1 proteins was significantly decreased in 9-ING-41-treated bladder cancer cells (Fig. 2A). Moreover, treatment with 9-ING-41 led to a decreased expression of antiapoptotic molecules, Bcl-2 and XIAP, resulted in an increased apoptosis as shown by PARP cleavage in bladder cancer cells (Fig. 2A,B). Furthermore, we used caspase activation assay to demonstrate that 9-ING-41 treatment induces apoptotic cell death in bladder cancer cells (Fig. 2C). Our *in vitro* results suggest that treatment with GSK-3 inhibitor 9-ING-41 suppresses expression of G2/M regulatory proteins and antiapoptotic molecules leading to cell cycle arrest and apoptosis in bladder cancer cells, and identify 9-ING-41 as a candidate for the targeted therapy of human bladder cancer.

### Inhibition of autophagy potentiates antitumor effects of 9-ING-41 in bladder cancer cells

**Figure 3:**
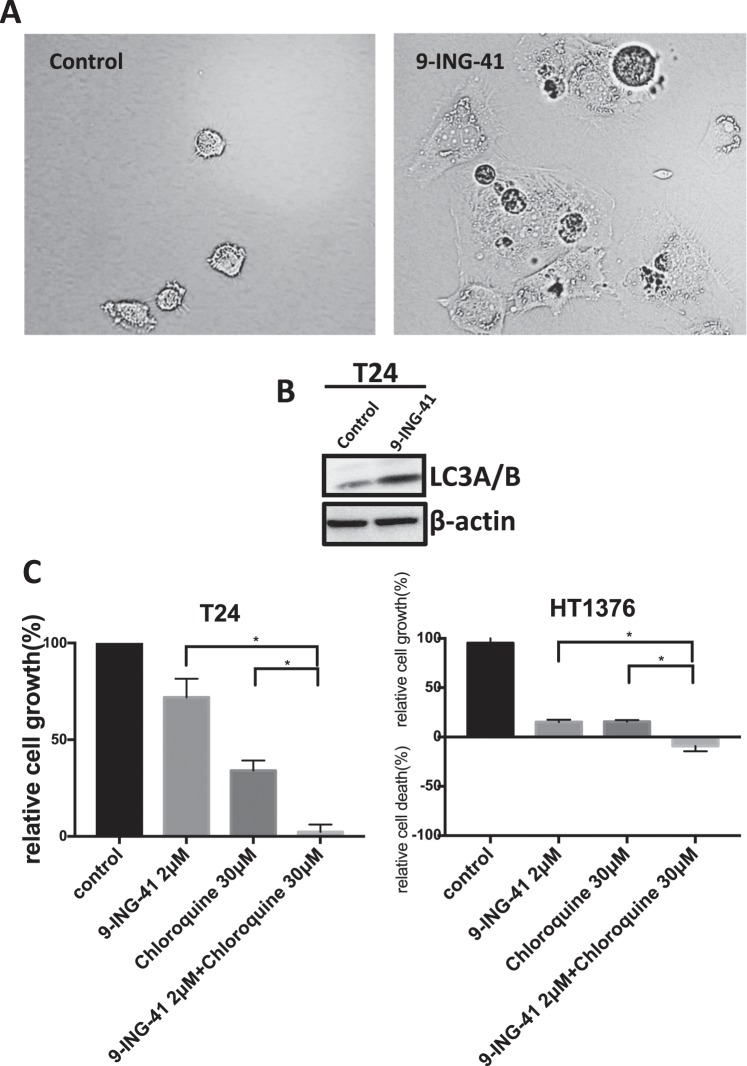
Inhibition of autophagy potentiates the antitumor effects of 9-ING-41 in bladder cancer cells. (**A**) Brightfield images of T24 cancer cells treated with Vehicle (Control) and 9-ING-41 5 μM for 24 hours. (**B**) T24 bladder cancer cells were treated with 9-ING-41 10 μM for 48 hrs. Post-treatment, whole cell lysate was prepared from the cells, separated by SDS-PAGE (20 μg/well), transferred to PVDF membrane, and immunoblotted as indicated. (**C**) Relative cell growth was measured by MTS assay in bladder cancer cell lines T24 and HT1376 treated with 9-ING-41 in combination with autophagy inhibitor chloroquine for 72 hours as indicated. Columns, mean; bars, SD. *P < 0.05 by one-way ANOVA with Tukey post-hoc test.

Despite the extensive investigation on the role of autophagy in tumor growth and metabolism, it is still unclear whether autophagy supports or inhibits viability of cancer cells^19^. In cancer cells, autophagy could be induced by different antitumor therapies^20–22^. We noticed significant morphological changes in 9-ING-41-treated bladder cancer cells (Fig. 3A). After 24 hours treatment with 9-ING-41, T24 cancer cells showed extensive vacuolation and formation of autophagosome like structures in the cytoplasm (Fig. 3A). The development of autophagy was confirmed by detection of an increased expression of LC3, an autophagy marker, in 9-ING-41-treated bladder cancer cells (Fig. 3B). Moreover, we found that combination of 9-ING-41 and chloroquine, an autophagy inhibitor, significantly suppressed viability of T24 (P < 0.05) and HT1376 (P < 0.05) cancer cells as compared to the effects of 9-ING-41 or chloroquine monotherapy (Fig. 3C). These results suggest that inhibition of autophagy could potentiate the antitumor effect of 9-ING-41 in bladder cancer cells.

### 9-ING-41 potentiates antitumor effect of gemcitabine and cisplatin in bladder cancer cells

**Figure 4:**
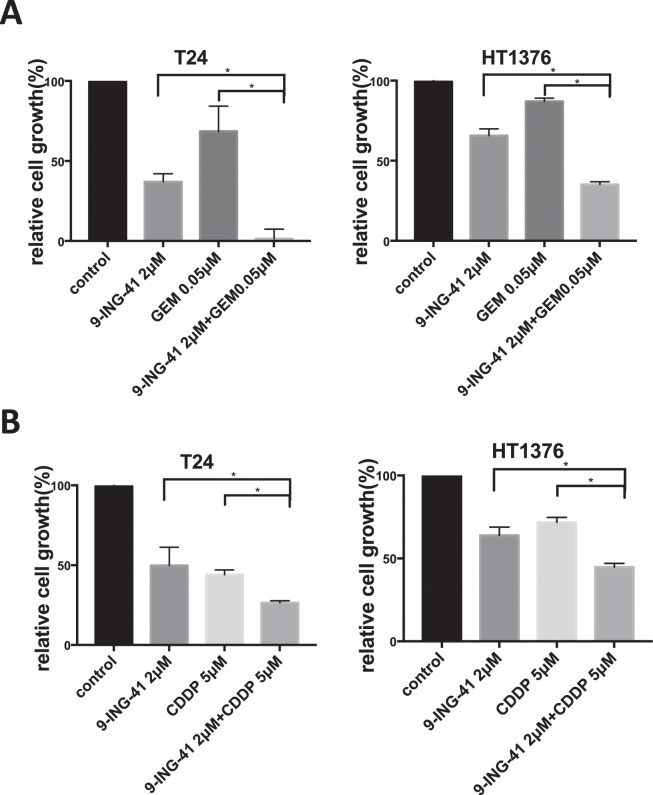
Pharmacological inhibition of GSK-3β with 9-ING-41 potentiates the effect of anticancer therapeutics in bladder cancer cells. (**A,B**) Relative cell growth was measured by MTS assay in bladder cancer cell lines T24 and HT1376 treated with 9-ING-41 in combination with gemcitabine (**A**) and cisplatin (**B**) for 3 hours as indicated. After the treatment, drugs were replaced with fresh culture medium and relative cell growth was measured by MTS assay after 72 hours. Columns, mean; bars, SD. *P < 0.05 by one-way ANOVA with Tukey post-hoc test.

It has been demonstrated that GSK-3 positively regulates NF-κB-mediated survival and chemoresistance of cancer cells^23^. Using chemoresistant p53-mut T24 and HT1376 bladder cancer cell lines, we tested our hypothesis that 9-ING-41 may overcome resistance to standard of care chemotherapeutic drugs in bladder cancer. Because the plasma half-life of 9-ING-41 is approximately 3 hours, we treated bladder cancer cells for 3 hours with 2 μM 9-ING-41 and chemotherapeutic drugs. Post-treatment, all test compounds were replaced with fresh cell culture medium. Then, bladder cancer cells were allowed to grow for 72 hours and relative cell growth was evaluated by MTS assay after 72 hours of cell incubation in drug-free culture medium. We found that 9-ING-41 potentiates the antitumor effects of gemcitabine (P < 0.05) and cisplatin (P < 0.05) in T24 and HT1376 bladder cancer cells (Fig. 4).

### 9-ING-41 therapy enhances cytotoxic effect of human immune cells

**Figure 5:**
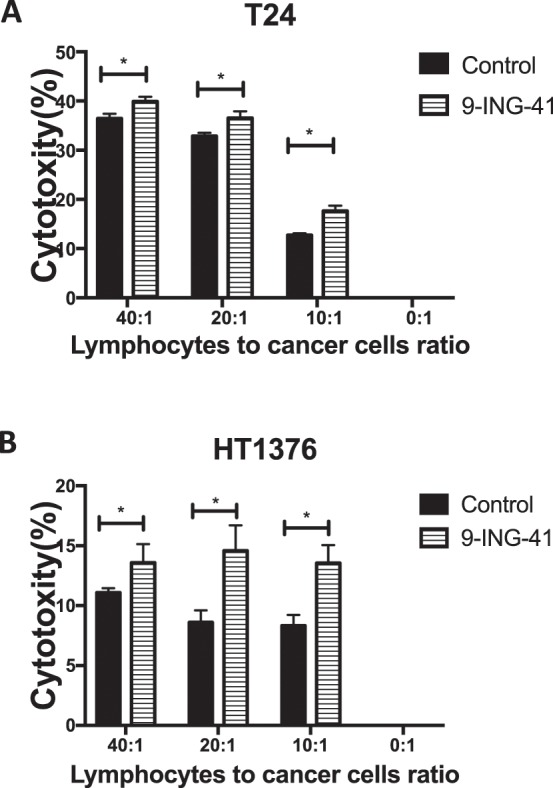
Treatment with 9-ING-41 sensitizes bladder cancer cells to cytotoxic effects of human immune cells. T24 (**A**) and HT1376 (**B**) bladder cancer cells were treated with 9-ING-41 for 48 hrs as indicated, harvested and mixed with human immune cells. LDH activity of supernatant was measured for evaluation of cytotoxicity. Columns, mean; bars, SD. Statistical analysis was performed using unpaired t-test. *P < 0.05.

To explore whether GSK-3 inhibition potentiates cytotoxic effect of human immune cells in bladder cancer cell lines, we treated T24 and HT1376 bladder cancer cell lines with 9-ING-41 for 48 hours, added the LAK cells at various ratio and measured LDH activity of supernatant to evaluate a cytotoxic effect of immune cells. Our results demonstrate that treatment with 9-ING-41 significantly increased cytotoxic effect of human immune cells in bladder cancer cell lines (Fig. 5).

## Discussion

GSK-3β has been identified as a potential therapeutic target in human bladder cancer^11^. Pharmacologic inhibition or genetic depletion of GSK-3β resulted in a decreased viability of bladder cancer cells^11^. In the present study, we tested the GSK-3β inhibitor 9-ING-41, a targeted therapeutic currently being evaluated in a phase 1/2 clinical trial in advanced cancer patients, in bladder cancer cell lines. This is the first report of 9-ING-41 efficacy in bladder cancer. This compound previously demonstrated significant antitumor activity *in vitro* and *in vivo* in pre-clinical models of neuroblastoma^14^, lymphoma^15^, glioblastoma^16^, ovarian^12^, pancreatic^17^, renal^10^ and breast^18^ cancer.

Recently it has been demonstrated that genetic depletion or pharmacologic inhibition of GSK-3β by 9-ING-41 induced cell cycle arrest at G2/M in lymphoma cells^24^. Consistent with the results from the lymphoma study^24^, we found cell cycle blockage at G2/M in 9-ING-41-treated bladder cancer cells, suggesting that GSK-3 inactivation by 9-ING-41 halts progression of mitosis in bladder cancer cells. The exact mechanism by which GSK-3 supports progression of mitosis in cancer cells is unknown. Here, we identified that expression of cyclin B1 and Cdk1, cell cycle regulatory proteins, are affected by GSK-3 inhibition in cancer cells. It has been shown that cyclin B1–Cdk1 complex is a key regulator of mitotic entry^25^. A large number of proteins are phosphorylated by the cyclin B1–Cdk1 complex prior to mitotic entry^25^. We found that expression of cyclin B1 and Cdk1 are downregulated in bladder cancer cell lines treated with 9-ING-41. Our results suggest that GSK-3 positively regulates the expression of cyclin B1 and Cdk1, and treatment with 9-ING-41 leads to cell cycle arrest at G2-phase in bladder cancer cells.

It has been shown that inhibition of GSK-3β leads to apoptosis via p53 activation in p53-wt colon cancer cells^26,27^. Although GSK-3β depletion didn’t affect p53-null colon cancer cell lines, it has been demonstrated that GSK-3β silencing sensitized chemoresistant p53-null colon cancer cells to antitumor effects of DNA–damaging drugs^26^. In agreement with the results of colon cancer studies^26^, we found that the GSK-3 inhibitor 9-ING-41 induced apoptosis in p53-wt RT4 bladder cancer cell line, whereas p53-mut HT1376 bladder cancer cells were more resistant to 9-ING-41 therapy which showed mostly growth inhibitory effect. These results suggest that GSK-3 targeted therapy with 9-ING-41 might be an effective treatment for p53-wt bladder cancer. GSK-3β has been shown as a positive regulator of NF-κB-mediated chemoresistance of cancer cells^23^. Here, we demonstrate that GSK-3 inhibitor 9-ING-41 potentiates the antitumor effect of conventional chemotherapeutic drugs in chemoresistant p53-mut T24 and HT1376 bladder cancer cells. Our results identify 9-ING-41 as a candidate for the treatment of p53-wt and p53-mut bladder cancer, and support a rationale to combine 9-ING-41 with gemcitabine or cisplatin, standard of care chemotherapeutic drugs, for metastatic bladder cancer therapy.

Whether autophagy contributes to cancer cell death or represents a mechanism of resistance to anticancer therapy remains unclear^19^. Recent studies demonstrated that GSK-3 inhibits autophagy through the mammalian target of rapamycin (mTOR) complex 1 (mTORC1)^28^. It has been shown that inhibition of GSK-3 decreased mTORC1 activity and increased autophagic flux in cancer cells^28^. Moreover, overexpression of either GSK-3*α* or GSK-3*β* activates mTORC1 and suppresses autophagy in breast cancer cells^28^. It has been demonstrated that inhibition of GSK-3 induced an autophagic response in pancreatic^29^, prostate^30^ and renal cancer^10^. In agreement with previous studies, we found that treatment with 9-ING-41 resulted in autophagy in bladder cancer cells. Importantly, we demonstrated that inhibition of autophagy with chroloquine potentiates the antitumor effect of 9-ING-41 in bladder cancer cells. Our results suggest that potential autophagy-mediated resistance to GSK-3 inhibitor 9-ING-41 could be overcome by using a combination of 9-ING-41 with autophagy inhibitor in human bladder cancer.

Cisplatin-based chemotherapy remains the standard treatment in metastatic bladder cancer patients. Recently, immune checkpoint inhibitors pembrolizumab, atezolizumab, durvalumab, nivolumab and avelumab were approved by FDA for the second line setting in metastatic bladder cancer patients who failed cisplatin-based chemotherapy^31^. In the present study, we found that treatment with GSK-3 inhibitor 9-ING-41 enhanced the cytotoxic effect of human immune cells in bladder cancer cell lines. Previously published studies demonstrated that GSK-3 inhibition could lead to upregulation of Fas ligand in cancer cells^32–34^ which could potentially enhance the sensitivity of cancer cells to cytotoxic effects of human immune cells. Our results demonstrate that 9-ING-41-treated bladder cancer cells are more sensitive to activated human immune cells. Our results suggest that 9-ING-41 therapy might potentiate antitumor immune response in bladder cancer patients.

Overall, the results of the current study provide evidence that treatment with 9-ING-41 could be a potential therapeutic approach to overcome tumor chemo- and immune-resistance in bladder cancer and provide a rationale for the inclusion of patients with advanced bladder cancer in clinical studies of 9-ING-41.

## Methods

### Cell culture and materials

Bladder cancer cell lines T24, HT1376, and RT4 were obtained from the American Type Culture Collection. Cells were cultured in RPMI 1640 (Gibco Invitrogen, Grand Island, NY) supplemented with 10% fetal bovine serum (FBS), 1% MEM nonessential amino acids, 1% MEM sodium pyruvate solution 100 mM, 0.14% NaHCO_3_, and 80 mg/L of kanamycin, at 37 °C in a humidified 5% CO_2_ atmosphere. 9-ING-41 was provided by Actuate Therapeutics, Inc. (Fort Worth, TX).

### Measurement of cell viability and cell proliferation

Cell viability was measured with a colorimetric CellTiter 96 AQueous One Solution Cell Proliferation Assay (Promega, Madison, WI) according to the manufacturer’s instructions as described previously^11^. BrdU cell proliferation assay (Calbiochem, Darmstadt, Germany) was carried out according to the manufacturer’s instructions^11^. Experiments were performed in three replicates using a flat-bottom 96-well plate (Corning, NY). GI_50_, a concentration of the drug that inhibits the growth of cancer cells by 50%, was calculated using GraphPad Prism 7 (GraphPad, San Diego, CA).

### RNA extraction and RT-PCR

Total cellular RNA was extracted using the SV total RNA Isolation System (Promega, Madison, WI) and first-strand DNA was synthesized using a High Capacity cDNA Reverse Trascription Kit (Applied Biosystems, Waltham, MA) according to the manufacturer’s instructions as described previously^11^. Real-time quantitative reverse transcriptase-PCR (RT-PCR) was done in the 7500 Real Time PCR System (Applied Biosystems, Waltham, MA) using pre-designed TaqMan Universal PCR Mastermix (Applied Biosystems, Waltham, MA) targeting human Bcl-2 and XIAP mRNA, and GAPDH was used as endogenous control^11^. Each experiment was repeated three times to confirm reproducibility with the reaction in triplicate wells for each sample. The expression of the target mRNA was quantified relative to that of the GAPDH mRNA^11^.

### Western immunoblotting analysis

We performed immunoblotting analysis as described previously^11^. The following antibodies were used: GSK-3β, Cyclin B1, Cdk1, β-actin from Cell Signaling Technology (Danvers, MA); XIAP, PARP from BD Biosciences (Franklin Lakes, NJ). We comply with the digital image and integrity policy.

### Cell cycle analysis

Cancer cells were cultured in medium with 9-ING-41 for 72 hours. Cells were then harvested by trypsinization and fixed in ice-cold 70% ethanol for 30 minutes in 4 °C. The fixed cancer cells were washed by PBS twice and centrifuged at 150 g. Then cells were resuspended in a 500 μl of FxCycle PI/RNase Staining Solution (Life Technologies, Carlsbad, CA). Cell cycle analysis was performed using flow cytometry system (FACscan flow cytometer: Becton-Dickinson). G0-G1, S, and G2-M phases of the cell cycle were determined with FCS Express software.

### Caspase activation assay

Caspase activation was detected by measuring the activity of caspase-3 (DEVDase) using CaspACE Assay System, Colorimetric (Promega, Madison, WI). According to manufacturer’s instruction, labeled p-nitoroaniline (pNA) which was released from the substrate upon cleavage by DEVDase, was measured by a spectrophotometer at 405 nm.

### *In vitro* cytotoxic assay

The study was approved by Niigata University Ethical Committee(IRB No. 2620) and was carried out in accordance to the ethical principals of the Declaration of Helsinki. After obtaining informed consent, blood samples were obtained from healthy human volunteers. Peripheral blood mononuclear cells (PBMCs) were separated using Lymphocyte Separation Solution (Nacalai Tesque, Kyoto, Japan). For lymphokine-activated killer (LAK) induction, PBMCs were suspended at a concentration of 2 × 10^6^ cells/ml in RPMI 1640 containing 5% FCS and IL-2 was added at a concentration 2000 U/ml. PBMCs were cultured in 25-cm_2_ tissue culture flasks for 4–6 days at 37 °C in a 5% CO_2_ atmosphere. T24 and HT1376 cells were treated with 2.5 μM of 9-ING-41 for 48 hours. Untreated cells were used as control. Cytotoxic assay was done using the CytoTox 96 Non-Radioactive Cytotoxic Assay (Promega, Madison, WI) according to the manufacturer’s instructions. Released lactate dehydrogenase (LDH) was measured at 490 nM using IMARK microplate reader (Bio-Rad Laboratories, Inc., Tokyo, Japan).

### Statistical analysis

Continuous variables are presented as the mean ± standard deviation (SD). Cell viability assay data were analyzed using one-way ANOVA. Statistical analysis was performed using GraphPad Prism 7.0 software. P < 0.05 was considered statistically significant.

## Data Availability

All data generated or analyzed during this study are included in this manuscript.
